# Improved surface-enhanced Raman and catalytic activities of reduced graphene oxide–osmium hybrid nano thin films

**DOI:** 10.1098/rsos.170353

**Published:** 2017-09-06

**Authors:** C. Kavitha, K. Bramhaiah, Neena S. John, Shantanu Aggarwal

**Affiliations:** 1 Department of Chemistry, Centre for Advanced Materials Research, B.M.S. Institute of Technology, Avalahalli, Yelahanka, Visvesvaraya Technological University, Bangalore, Karnataka 560064, India; 2 Centre for Nano and Soft Matter Sciences, Bangalore, Jalahalli 560013, India; 3 Chemistry and Physics of Materials Unit, Jawaharlal Nehru Center for Advanced Scientific Research (JNCASR), Jakkur PO, Bangalore, Karnataka, India

**Keywords:** reduced graphene oxide, osmium, hybrid thin film, liquid/liquid interface method, catalytic activity, surface-enhanced Raman spectroscopy

## Abstract

Reduced graphene oxide–osmium (rGO-Os) hybrid nano dendtrites have been prepared by simple liquid/liquid interface method for the first time. The method involves the introduction of phase-transfered metal organic precursor in toluene phase and GO dispersion in the aqueous phase along with hydrazine hydrate as the reducing agent. Dendritic networks of Os nanoparticles and their aggregates decorating rGO layers are obtained. The substrate shows improved catalytic and surface-enhanced activities comparable with previous reports. The catalytic activity was tested for the reduction of *p*-nitroaniline into *p*-phenyldiamine with an excess amount of NaBH_4_. The catalytic activity factors of these hybrid films are 2.3 s^−1^ g^−1^ (Os film) and 4.4 s^−1^ g^−1^ (rGO-Os hybrid film), which are comparable with other noble metal nanoparticles such as Au, Ag, but lower than Pd-based catalysts. Surface-enhanced Raman spectroscopy (SERS) measurements have been done on rhodamine 6G (R6G) and methylene blue dyes. The enhancement factor for the R6G adsorbed on rGO-Os thin film is 1.0 × 10^5^ and for Os thin film is 7 × 10^3^. There is a 14-fold enhancement observed for Os hybrids with rGO. The enhanced catalytic and SERS activities of rGO-Os hybrid thin film prepared by simple liquid/liquid interface method open up new challenges in electrocatalytic application and SERS-based detection of biomolecules.

## 1. Introduction

Nanotechnology is an important field of modern research dealing with design, synthesis and manipulation of nanostructured materials ranging approximately from 1 to 100 nm. Nanofabrication via self-assembly of hybrid materials into well-defined architectures is essential for the next generation of miniaturized sensors. The targeted types of application mainly depend on the properties and functionality of the hybrid materials, which are determined by their chemical composition as well as morphological and structural parameters. Hence, synthesis of high performance and compatible nanohybrid thin films with desired properties via simple chemical methods is gaining significance in recent times. We have been using reduced graphene oxide (rGO)-based metal and metal oxide nanoparticles in the form of thin films prepared via liquid/liquid interface for applications involving surface-enhanced Raman spectroscopy (SERS)-based molecular detection [1,2]. We have shown that the charge transfer and fluorescence quenching by rGO aids in improved enhancement factors and detection of dyes. A synergic effect due to rGO and the hybrid counterpart is observed in the performance of the multifunctional material. In this article, we describe the use of rGO-Os hybrid thin films obtained at a liquid/liquid interface, as SERS substrates for dyes as well as catalytic activity. Osmium is the densest silvery metal, which is used in a few alloys and in industry as a catalyst. The metal is a strong oxidant and reacts with combustible and reducing materials. There are few reports available where Os particles were prepared by sophisticated physical vapour deposition routes [3–6] and in most cases, they are combined with other metals like Pd or Pt. The application of Os thin films appears in the patent literature only related to the fields of fuel cells, catalysis, sensors and electronic devices [5,7,8]. There is a need to explore simple synthetic procedures to obtain different Os nanostructures and their graphene hybrids under environmentally friendly conditions in turn to study their optical properties through surface plasmon absorption. Only a few reports exist on the synthesis and properties of Os nanostructures; especially rGO-Os hybrid materials have not been reported yet in any of the synthesis methods. Hence, we have sought to synthesize Os and rGO-Os films by simple chemical methods and explore their applications. The films exhibit absorptions in visible and near IR regions. We show that Os nanostructures, when coupled with rGO, have superior catalytic and Raman enhancement factors. The mechanism is discussed in this paper.

## 2. Experimental methods

### 2.1. Synthesis of osmium and reduced graphene oxide–osmium thin films

Hybrid films of Os nanoparticles (NPs) and rGO-Os NPs have been prepared employing the liquid/liquid interface method involving interfacial *in situ* reduction of the metal organic precursor in toluene and GO in the aqueous phase. Exfoliated graphite oxide was prepared from graphite particles (−300 mesh, Alfa-Aesar) by adopting a modified Hummers method [9]. A standard solution of GO was prepared and sonicated for further use. Osmium(III) chloride was received from Aldrich.

OsCl_3_ (1 ml, 25 mM in 25 ml deionized water) was phase transferred to toluene containing 100 µmol of tetraoctylammonium bromide by vigorous shaking in a separating funnel. After 30 min, toluene phase changed from colourless to brownish red, indicating successful phase transfer. The organic phase was separated and poured over the aqueous phase (25 ml) containing 3 mg GO; 50 µl of hydrazine hydrate was injected to the aqueous phase with minimal disturbance and the whole system was heated at 90°C in an oil bath for 90 min. After 1 h an ultra-thin film of rGO-Os NPs was observed at the liquid/liquid interface. Bare Os films were also obtained by the same method except for the addition of GO.

### 2.2. Characterization

UV–visible–NIR spectra were recorded using a PerkinElmer Lambda 750 spectrophotometer. For this, rGO-Os NP films were collected on quartz plates or dispersed in Milli-Q water by sonication. Field emission scanning electron microscopy (FESEM) images and energy dispersive X-ray spectroscopy (EDS) spectra were obtained using TESCAN MIRA3 LM (Czech Republic) and QUANTAX EDX (Bruker) instruments. X-ray diffraction studies of as-synthesized composites were done using a Rigaku SmartLab diffractometer equipped with parallel beam optics, and Cu Kα radiation (*kα* = 1.54 Å, 40 kV, 30 mA) was incident at a grazing angle of 0.3°.

### 2.3. Catalysis

For catalysis studies, freshly prepared aqueous solutions of 4-nitroaniline (1.5 ml, 0.2 mM) and NaBH_4_ (1 ml, 100 mM) were mixed in a 3 ml quartz cuvette followed by the addition of Os and rGO-Os NPs hybrid film (0.1–0.15 mg) collected on a glass substrate. The bright yellowish green colour became colourless during the course of the reaction. The progress of the reaction was monitored *in situ* in a spectrophotometer by recording the UV–visible spectra at regular intervals of time in the range of 200–500 nm at room temperature.

### 2.4. Surface-enhanced Raman scattering instrumentation

For SERS studies, rhodamine 6G (R6G) and methylene blue (MB) fluorescent dyes were used as the analytes. Ethanolic solution of R6G with various molar concentrations was drop casted onto Os films and rGO-Os hybrid thin films collected on glass substrates. SERS measurements were performed with a home-built Raman spectrometer equipped with a solid-state diode-pumped Nd-YAG laser (Coherent Inc.) of power approximately 120 mW. The excitation wavelength of 532 nm was focused onto the sample through a modified epi-illuminator of a 50i Nikkon microscope. A 50× microscope objective having numerical aperture 0.45 was used to focus the incident light as well as collect the scattered light in a 180° backscattering geometry. A 100 µm single core, multimode optical fibre was used to collect the scattered light from the microscope through a dichroic beam splitter (660DCLP, Chroma Technology Corp.). The details of the set-up are given elsewhere [10]. For Rayleigh rejection, the scattered light was passed through an edge filter (Semrock), which was placed before the optical fibre. The Raman spectra were recorded using a Jobin-Yvon Triax 550 with a liquid N_2_ cooled CCD detector (Horiba). A digital camera (Nikon Coolpix5400, Nikon) on top of the microscope was used to check the focus of the laser light on the sample and to align it to the optical fibre. The laser power was measured to be around 30 mW near the sample. The typical accumulation time for each spectrum was 30 s.

## 3. Results and discussion

**Figure 1.**
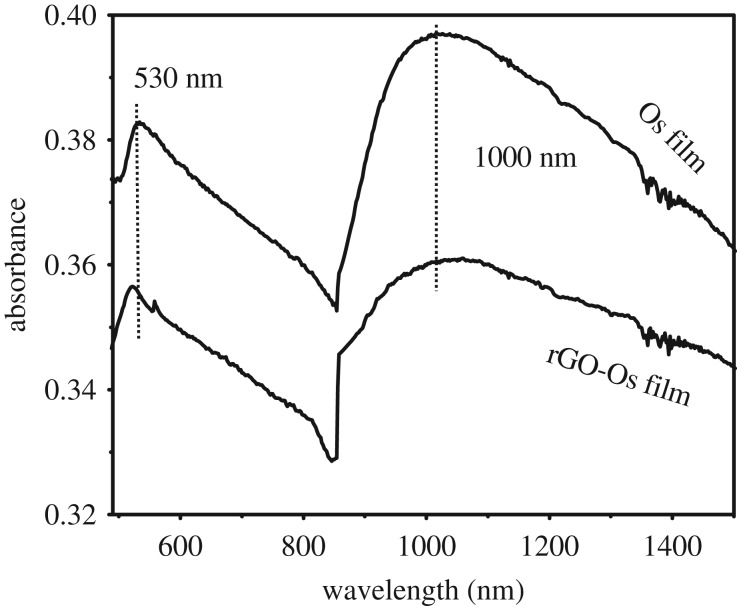
UV–visible–NIR absorption spectra of as-synthesized Os and rGO-Os NP hybrid films supported on glass, obtained by liquid/liquid interface method.

The Os NP and rGO-Os NP films show broad coupled surface plasmon resonance (SPR) bands around 530 nm and 850–1100 nm range as indicated in figure 1. This is due to the presence of ultra-small NPs aggregated into clusters giving rise to particle–particle interaction. The SPR bands of isolated Os clusters are reported at approximately 350 nm while for interconnected Os nanochains, SPR bands in the visible region (540 nm) have been observed [11,12]. The aggregation of small metal NPs causes the red shift in the SPR bands because of electric dipole–dipole interaction leading to the coupling between the plasmon oscillations of various ultra-small Os NPs. In the case of films, the interparticle interactions are stronger than in the case of a solution, and hence the surface plasmon coupling will be stronger to cause a large red shift with broadening of the SPR bands. This has been reported in the case of gold nanocrystalline films obtained at a liquid/liquid interface [13]. The broad absorption band centred around 1000 nm for Os and rGO-Os nanocrystalline films could also be due to coupled plasmonic interactions.

**Figure 2.**
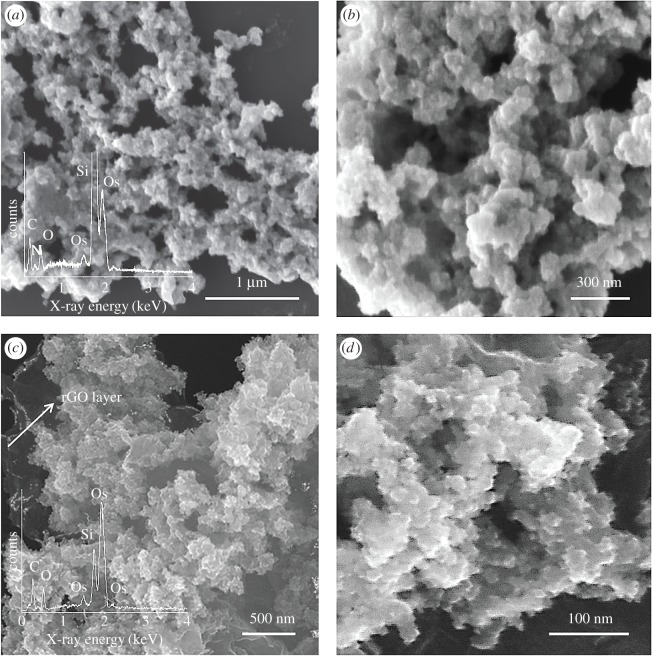
FESEM images and EDS analyses of hybrid films synthesized at a liquid/liquid interface. (*a*) Os NPs hybrid film and (*b*) rGO-Os NPs hybrid film.

The surface morphology and composition of ultrafine Os and rGO-Os NPs hybrid films have been studied by FESEM and EDS analysis. Figure 2*a* shows the Os NP film, in which the Os NPs are seen aggregated to form interconnected dendritic network extended to large scale area. The inset gives the EDS spectra, showing Os species. Si signal is from the substrate. Figure 2*b* shows the higher magnification image of the Os aggregates. In the case of rGO-Os NP hybrid films (figure 2*c*), Os NP clusters are seen interspersed with rGO layers. The EDS spectral analysis in the inset confirms the presence of Os NPs on the rGO sheets. The higher magnification image given in figure 2*d* shows the Os aggregates on rGO.

**Figure 3.**
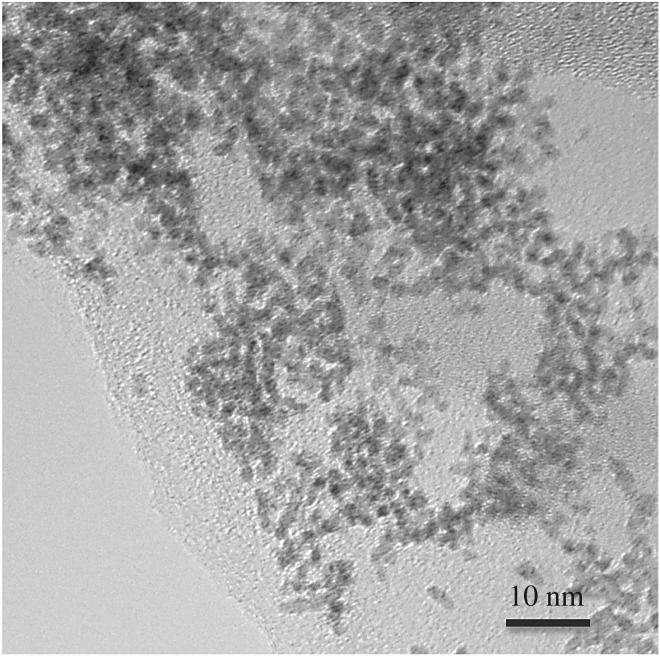
Transmission electron microscopy image of rGO-Os hybrid film.

The transmission electron microscopy image of rGO-Os NP film given in figure 3 reveals that the Os aggregates consist of 2–3 nm Os NPs interconnected and interspersed with rGO layers clearly.

### 3.1. Catalysis

**Figure 4.**
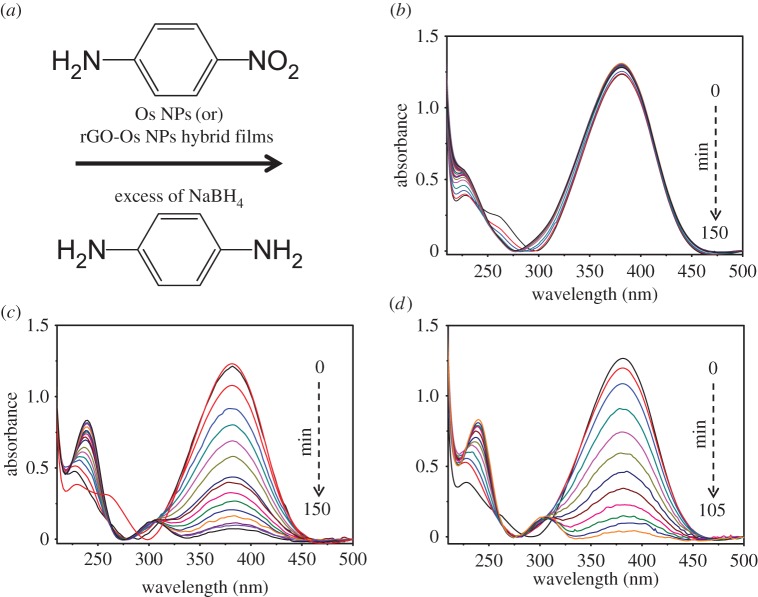
(*a*) Reaction scheme for the conversion of *p*-NA into *p*-PDA by borohydride in the presence of Os NP and rGO-Os NP hybrid films. UV–visible spectra acquired during the conversion of *p*-NA into *p*-PDA with an excess amount of NaBH_4_ solution: (*b*) uncatalysed reaction, (*c*) in the presence of Os NP film and (*d*) with rGO-Os NP hybrid film.

The catalytic activity of Os NPs and rGO-based Os NPs hybrid films manufactured employing liquid/liquid interface was tested by taking a model reaction: reduction of *p*-nitroaniline (*p*-NA) into *p*-phenyldiamine (*p*-PDA) with an excess amount of NaBH_4_ (figure 4*a*). There are several reports on the reduction of aromatic nitro compounds with noble metal NPs [14], but only a handful of reports on Os NPs as the catalyst [15,16]. The reaction can be monitored by UV–visible spectroscopy because *p*-NA shows distinctive absorption peak at 380 nm and the product *p*-PDA displays weak absorption peaks around 239 and 300 nm. Figure 4*b* shows that in the absence of the catalyst, the peak height remained unchanged with time. Figure 4*c,d* shows the UV–visible spectra acquired during the reduction of *p*-nA into the *p*-PDA in the presence of Os NP films and rGO-Os hybrid films. When the Os and rGO-Os NPs films supported on the glass substrate are added to the reaction system, the reduction reaction got initiated. The progress of the reaction is visualized as a decrease in 380 nm absorption intensity and increase in 239 and 300 nm absorption due to the formation of *p*-PDA in the solution. The rate of reaction is considered to be independent of NaBH_4_ because an excess amount is taken. The peak at 380 nm decreases progressively with time and it takes 150 min for the completion of the reaction in the presence of Os NP film while only 105 min is required in the presence of rGO-Os NP hybrid film. BH_4_^−^ adsorbs on Os surface and initiates the reaction by transferring a surface hydrogen species (hydride). At the same time, *p*-NA molecules also adsorb on the surface of the catalyst and get reduced by the surface hydrogen species to yield the *p*-PDA [15,17].

When the Os and rGO-Os NPs films on the glass substrate are added to the reaction system, the reduction reaction started as depicted in figure 4*c*,*d*. The peak at 380 nm decreases progressively with time and it takes 150 min for completion of the reaction in the presence of Os NP film while only 105 min is required in the presence of rGO-Os NP hybrid film.

**Figure 5.**
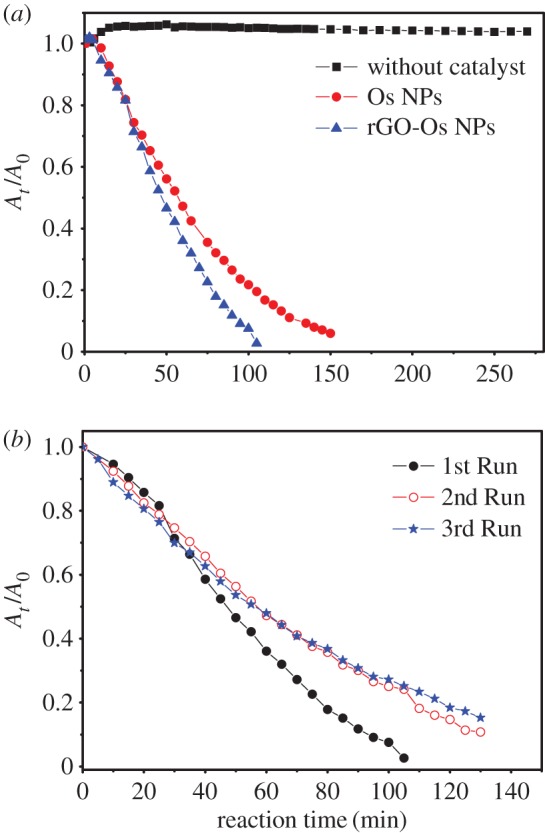
(*a*) Reduction kinetics of *p*-NA by sodium borohydride in the absence of any catalyst and in the presence of Os NPs and rGO-Os NPs hybrid films. (*b*) Recyclability of rGO-Os NPs hybrid film for three runs.

The kinetics of the reaction in the absence and presence of catalyst was studied by plotting the absorbance (at 380 nm) versus time given in figure 5. The absorbance is taken as proportional to the concentration of *p*-NA. The reaction follows pseudo first-order kinetics and the rate expression is given as

3.1 $$[A_{t}]=[A_{0}]\;e^{-kt},$$

where *A_t_* and *A*_0_ are the absorbance values at a particular time *t* during the reaction and that at the start of the reaction after the addition of catalyst (considered zero time) and *k* is the observed apparent rate constant. The apparent rate constant (*k*) value has been estimated by exponential fitting using equation (3.1). The apparent rate constant of the Os NPs and rGO-Os NPs hybrid films is 2.5 × 10^−4^ s^−1^ and 4.83 × 10^−4^ s^−1^, respectively. The catalytic activity factors (*k* = *K*/*w*, where *w* is the weight of the sample) of these hybrid films are 2.3 s^−1^ g^−1^ (Os film) and 4.4 s^−1^ g^−1^ (rGO-Os hybrid film), which are comparable with other noble metal NPs such as Au, Ag, but lower than Pd-based catalysts [14]. The usage of these films on substrates allows easy recovery of the catalyst and the recyclability for three runs has been monitored (figure 5*b*). A slight decrease in the rate is noted. It is well known that in a homogeneous catalysis reaction, the catalytic rate depends mainly on effective surface area available. Normally, the higher catalytic rate for aggregated clusters might be due to their overall increase in size due to aggregation. When these Os dendrites combine with rGO, the effective surface area is increased as compared to Os dendrites alone. Owing to the increase in effective surface area, the electron transfer from the reducing agent to the nitro compound becomes more feasible and faster which in turn increases the catalytic rate tremendously. So the synergic effect of combined rGO-Os hybrid enhances the catalytic reactions.

### 3.2. Surface-enhanced Raman spectroscopy measurements

To explore the potential applications of as-synthesized Os NPs and rGO-Os NPs hybrid films as promising SERS-active substrates, we have examined Raman spectra of R6G and MB as fluorescent analytes adsorbed on these films.

**Figure 6.**
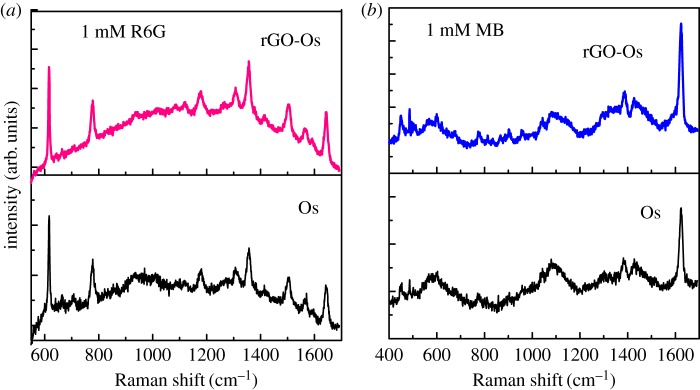
SERS spectra of (*a*) 1 mM R6G and (*b*) 1 mM MB on Os and rGO-Os films prepared by liquid/liquid interface method.

The Raman enhancement is clearly observed in figure 6 for 1 mM R6G and 1 mM MB on Os and rGO-Os thin film prepared by liquid/liquid interface method. The fluorescence background is seen slightly quenched in the presence of rGO. It has been reported previously that Os itself is a good SERS-active substrate for dyes [15,16]. Assignments of Raman bands for neat analytes and SERS for R6G and MB dyes are given in tables 1 and 2.

**Table 1.** Raman band assignment of R6G.

neat R6G experimental	experimental SERS of 1 mM R6G on rGO-Os hybrid films	assignment
610	611	in-plane C_x_─C_x_─C_x_ bending
768	780	C_x_─H out of plane bend
1182	1181	C_x_─C_x_ st
1192		C_e_─C_e_ st and C_e_─H bend
1313	1306	C_x_─H bend/C─N st
1361	1353	C_x_─C_x_ st/C─N st
	1416	C_e_─H bend
1512	1508	C_x_─C_x_ st
	1564	C_x_─C_x_ st
	1593	C_x_─C_x_ st
1650	1647	C_x_─C_x_ st

**Table 2.** Raman band assignment of MB.

neat MB (aqueous solution) experimental	experimental SERS of 1 mM MB on rGO-Os hybrid films	assignment
447	445	skeletal deformation
501	496	skeletal deformation
597	591	skeletal deformation
1042	1040	in-plane bending (C─H)
	1091	in-plane bending (C─H
1187		C─N stretching
1394	1385	in-plane ring deformation (C─H)
1442	1441	asymmetric (C─N) ring stretching
1468		asymmetric (C─C) ring stretching
1500	1503	stretching (C─C) ring
1624	1623	stretching (C─C) ring

**Figure 7.**
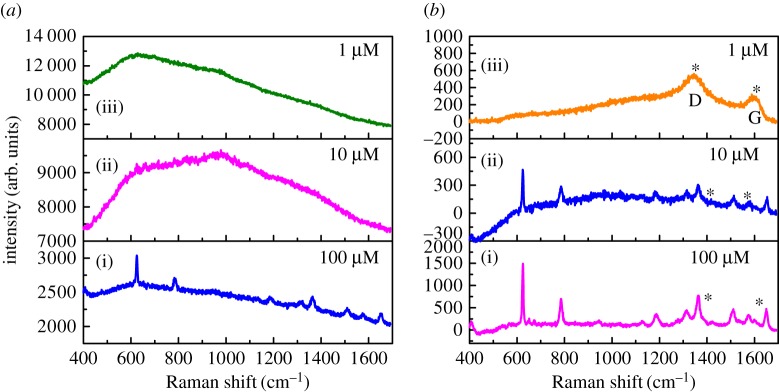
SERS spectra of various concentrations (i) 10^−4^, (ii) 10^−5^, (iii) 10^−6^ M methanolic solution of R6G adsorbed on (*a*) Os and (*b*) rGO-Os hybrid nanostructured films under 532 nm laser excitation. The asterisks show the D band (1345 cm^−1^) and G band (1596 cm^−1^) from rGO.

SERS activity of the films for various concentrations of the analyte has also been studied and is given in figure 7. It is clear from figure 7*a* that the Raman profile of R6G has a fluorescent background for Os NPs substrate and can be detected to 10^−4^ M only. But in the case of rGO-Os (figure 7*b*), one can see the resolved spectra of R6G and the detection limit is 10^−5^ M, one order of magnitude higher than that for Os NP film. The presence of rGO is seen from the D (1345 cm^−1^) and G (1596 cm^−1^) bands in figure 7*b* denoted by asterisks. It is evident that the enhancement of each R6G Raman band is also higher compared to that obtained from Os substrate. Apart from the Raman enhancement of rGO-Os hybrid thin films, they are responsible for uniform signal on different points of the substrate and they are stable after 10 days also. The SERS mechanism could be due to (i) electromagnetic enhancement and (ii) chemical enhancement. Electromagnetic enhancement has supreme influence in the case of metal NPs because the oscillating electric field of a light beam induces an oscillating dipole in a metal particle where the excitation energy was localized in some specific places called ‘hot spots' instead of distributed uniformly on the particles. Further, the enhancement can be improved for the aggregation of NPs as compared to mono-dispersed spherical particles [18–20]. So in the case of osmium nanodentrites, the enhancement is due to the plasmonic coupling effect of NP aggregates creating a high local electromagnetic field enhancement and consequently strong Raman cross section on the hotspots. In the case of rGO-Os nanostructured thin film, it is well known that the pronounced shift of the Raman band in SERS arises from a change in the geometrical or electronic structure of the adsorbed molecules and may be attributed to the charge transfer mechanism or the direct chemical interaction between molecule and functional group of rGO [21]. Moreover, the sensitivity of rGO-Os substrate is rather determined by a combination of chemical enhancement of rGO and the number of ‘hot spots’ of Os dendtrites.

#### 3.2.1. Surface gain (*G*) factor calculation

The enhanced Raman signal can be quantitatively analysed by the estimation of enhancement (*G*) factor. This can be calculated as

3.2 $$\mathrm{EF}=\left(\frac{I_{\mathrm{SERS}}}{I_{\mathrm{bulk}}}\right)\left(\frac{N_{\mathrm{bulk}}}{N_{\mathrm{SERS}}}\right),$$

where *I*_SERS_ is the measured SERS intensity for the probe molecules on the NP surface, *I*_bulk_ is the measured intensity of normal Raman scattering from the bulk sample, *N*_bulk_ is the number of probe molecules under laser illumination in the bulk sample and *N*_SERS_ is the number of the molecules probed on the NP surface. Here, the enhancement factor is calculated for R6G Raman spectra with 532 nm laser excitation because the 530 nm SPR band is responsible for Raman enhancement. The prominent band at 611 cm^−1^ has been chosen for calculating *G*-factor. Since neat R6G is highly fluorescent, 1 mM R6G aqueous solution has been analysed for bulk Raman spectra. The ratio of *I*_SERS_/*I*_bulk_ calculated for R6G adsorbed on rGO-Os hybrid thin film is approximately 13 and the same for Os thin film is approximately 9. The calculation details of *N*_bulk_ and *N*_SERS_ are given elsewhere [1]. The *G*-factor for the rGO-Os thin film is 1.0 × 10^5^ and for Os thin film is 7 × 10^3^ which is approximately onefold less than previously reported with mercaptopyridine as an analyte and excitation laser wavelengths of 514, 633 nm [12]. But the EF for the rGO/Os substrate shows a 14-fold enhancement compared with the Os one prepared by liquid/liquid interface method. This enhancement is more compared with GO-decorated Ag dendrites [22]. So the additional SERS enhancement results from two major factors. One factor is the chemical enhancement caused by the rGO layer. The chemical enhancement of rGO is due to the π–π stacking of R6G which contains a benzene ring in its molecular structure, similar to that of graphene. When it is deposited on rGO-Os nano hybrid films, the vibrational signal of R6G is notably enhanced. The other factor is the large surface-to-volume ratio induced by the Os dendritic nanostructures [22,23]. This further helps increase the rGO–molecule interaction area. So the synergic effect of rGO-Os is also responsible for SERS effect.

## 4. Conclusion

Self-assembly of noble metal NPs along with rGO is the best route to achieve improved properties by synergic effect between the metal NPs and rGO. We demonstrate that liquid/liquid interface method provides a simple and good way to generate Os nanostructures on the rGO layers in the form of thin films. The method involves the introduction of phase-transfered metal organic precursor in toluene phase and GO dispersion in the aqueous phase along with hydrazine hydrate as the reducing agent. Dendritic networks of Os NPs and Os NP aggregates decorating rGO layers are obtained. The capability of the rGO-Os hybrid film has been analysed for catalysis and SERS applications. The catalytic rates of these hybrid films are comparable with other noble metal NPs. The SERS activities of R6G and MB dye have been investigated. The *G*-factor for the rGO-Os thin film is 1.0 × 10^5^ and for Os thin film is 7 × 10^3^ for R6G where rGO-Os hybrid shows 14-fold enhancement over that of Os and proves the superior performance of Os NPs combined with rGO.

## Data accessibility

This article has no additional data.
